# Transcription-independent and -dependent p53-mediated apoptosis in response to genotoxic and non-genotoxic stress

**DOI:** 10.1038/s41420-019-0211-5

**Published:** 2019-08-27

**Authors:** Cheng-Jung Ho, Ru-Wei Lin, Wei-Hua Zhu, Tsung-Kai Wen, Chieh-Ju Hu, Yi-Lin Lee, Ta-I Hung, Chihuei Wang

**Affiliations:** 1 Department of Orthopedics, Kaohsiung Medical University Hospital, 80708 Kaohsiung, Taiwan; 2 Graduate Institute of Food Safety Management, National Pingtung University of Science and Technology, 91201 Pingtung, Taiwan; 3 Department of Biotechnology, Kaohsiung Medical University, 80708 Kaohsiung, Taiwan; 4 School of Post-Baccalaureate Chinese Medicine, Tzu Chi University, 97004 Hualien, Taiwan; 5 Department of Medical Research, Kaohsiung Medical University Hospital, 80708 Kaohsiung, Taiwan

**Keywords:** Apoptosis, Stress signalling

## Abstract

We previously reported that p53-mediated apoptosis is determined by severity of DNA damage, not by the level of p53, in doxorubicin-treated prostate cancer cells. In addition to doxorubicin, our results here indicated that camptothecin and bortezomib, which are a topoisomerase 1 poison and a 26 S proteasome inhibitor, respectively, could also induce apoptosis in a p53-dependent manner in prostate cancer. Then, we examined whether p53-mediated apoptosis induced by genotoxic and non-genotoxic stress occur in the same or a different way. By using dominant negative p53 to compete with wild-type p53 in transcription activity, we demonstrated that p53-mediated apoptosis in response to doxorubicin- or camptothecin-induced genotoxic stress is transcription-independent. In contrast, p53-mediated apoptosis from bortezomib-induced stress is transcription-dependent. Interestingly, we also found that doxorubicin-induced p21 expression is activated by p53 in transcription-dependent manner, while camptothecin-induced p21 expression is p53-independent. We then investigated the p53 ratio of nucleus to cytosol corresponding to low and high dose doxorubicin, camptothecin, or bortezomib treatment. The results suggested that p53 translocation from cytoplasm to nucleus actively drives cells toward apoptosis in either transcription-dependent or -independent manner for responding to non-genotoxic or genotoxic stress, respectively.

## Introduction

p53 functions as a tumor suppressor, as supported by evidence that *TP53* germline mutations in Li-Fraumeni syndrome predispose to a variety of early-onset cancers^1^, while mice with *Trp53* knockout acquire tumors at high penetrance^2^. Correspondingly, *TP53* somatic mutations are frequently found in human tumors^3^ and metastatic cancers^4^. Mutation of *TP53* in many types of cancer is associated with poor patient prognosis^5^.

Functionally, p53 is a transcription factor forming a homo-tetramer to activate nearly 500 target genes mainly responsible for cell cycle arrest, cell senescence, DNA repair, metabolic adaptation, and cell death^6^. p53 protects the integrity of the genome by driving severely damaged cells toward death, thus performing its role of tumor suppression in vivo. In addition to tumor suppression, p53-mediated apoptosis also plays an essential role in cancer chemotherapy. Cancer cells with wild-type *TP53* demonstrate higher sensitivity than cancer cells with mutated *TP53* in response to chemotherapy agents, mainly DNA damage agents^7^. The p53 target genes for its DNA damage response (DDR) have been widely explored.

The target genes involved in cell cycle arrest and DNA repair are *p21*, *GADD45A*, *DDB2*, *FANCC*, and *XPC*, which can rescue cells from DNA damage caused by chemical agents or radiation^8,9^. p53 also upregulates many genes such as *PUMA*, *NOXA*, *BAX*, and *APAF* to promote cell death through apoptosis in DDR^8,9^. How the differential transcription control of p53 determines cell fate, survival, or death is an interesting issue. The promoter selectivity proposal claims that p53 binds to its response elements differentially by both post-translational modifications and interactions with cofactors to activate cell survival or apoptosis genes^10^. However, the expression profiles induced by p53 in response to DDR reveal that both cell cycle arrest and apoptosis genes are transcribed by the same conditions^11–14^. Instead of promoter selectivity, the level of p53 is also considered as a threshold to mediate the cell fate decision between growth arrest and apoptosis^15^.

Our recent study found that p53-mediated apoptosis only occurs in severe DNA damage induced by high concentrations of doxorubicin (DOX), and not with low DNA damage even with high levels of p53^16^. This result suggested that while p53 is essential, substantial genotoxic stress might be the determining factor for apoptosis. In contrast, the expression of p21 corresponds to the level of p53 in low DNA damage conditions and decreases with heavy DNA damage and the occurrence of apoptosis^16^. Thus, cell cycle arrest and apoptosis are possibly regulated by p53 via different mechanisms.

To address the above issue, we first explored p53-mediated apoptosis induced by agents other than DOX, including camptothecin (CPT) and bortezomib (BTZ), which are a topoisomerase 1 poison and a 26 S proteasome inhibitor, respectively^17,18^. Then we investigated how p53 regulates responses to cellular stresses induced by DOX, CPT, or BTZ. Just like DOX, CPT and BTZ efficiently induced apoptosis in a p53-dependent manner in prostate cancer. By using dominant negative p53 (p53DN), p53mt135, to compete with wild-type (WT) p53 in transcription activity in prostate cancer, we demonstrated that p53-mediated apoptosis in response to DOX- or CPT-induced genotoxic stress is transcription-independent. In contrast, p53-mediated apoptosis for BTZ-induced stress is transcription-dependent. The p21 expression induced by DOX was transcription-dependent through p53, and nevertheless the p21 expression induced by CPT was p53-independent. Moreover, we investigated the p53 nucleus to cytosol ratio corresponding to low and high concentrations of DOX, CPT, or BTZ. We found that p53 translocation from cytoplasm always occurs no matter whether the cells enter cell cycle arrest or apoptosis.

## Results

### p53 regulated CPT-induced apoptosis

**Fig. 1:**
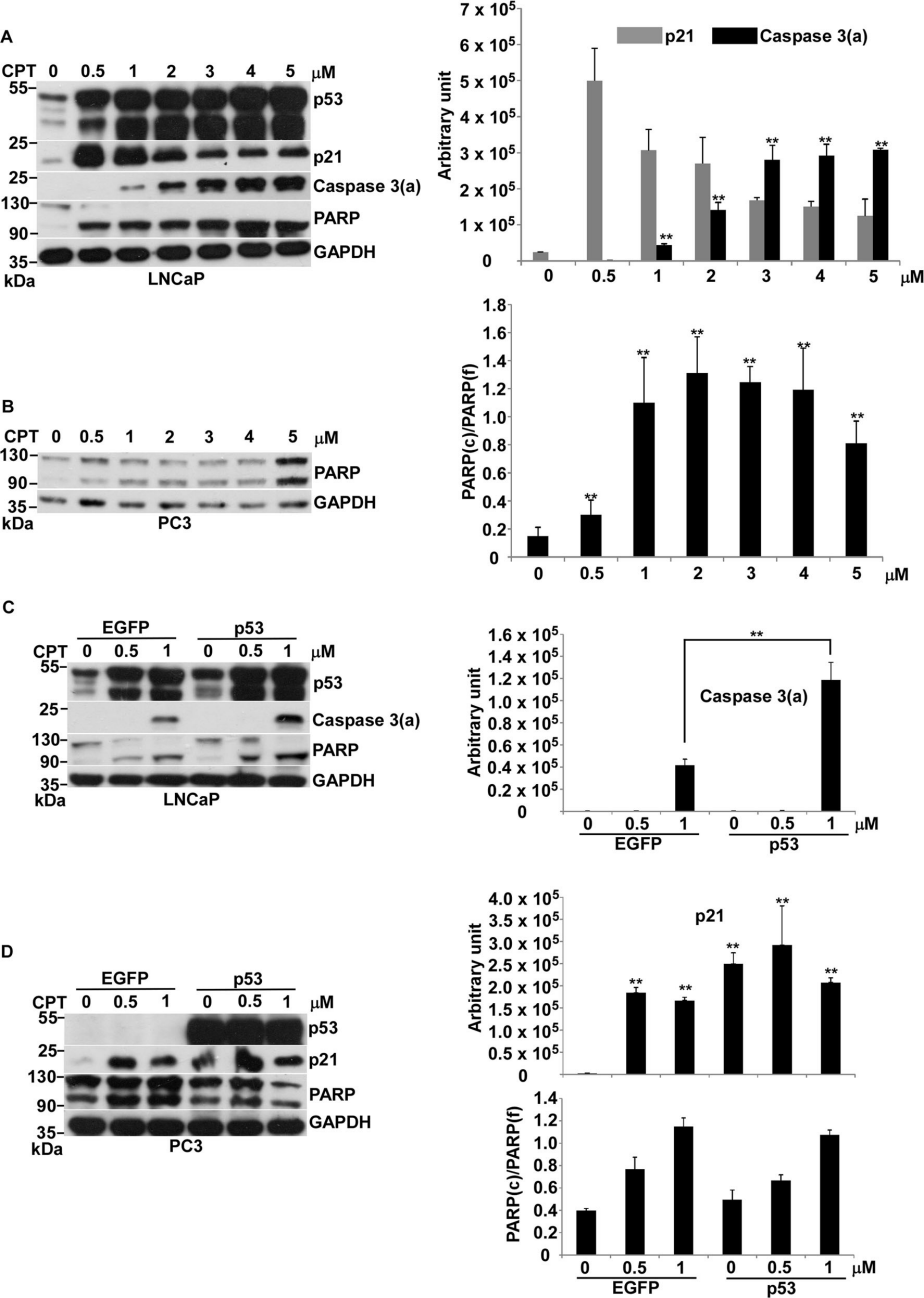
Effects of CPT on apoptosis in LNCaP cells. **a** Protein levels of p53, p21, caspase 3(a), PARP, and GAPDH in LNCaP cells treated with 0, 0.5, 1, 2, 3, 4, and 5 μM of CPT for 24 h was analyzed by immunoblotting. **b** Protein levels of PARP and GAPDH in PC3 cells treated with 0, 0.5, 1, 2, 3, 4, and 5 μM of CPT for 24 h was analyzed by immunoblotting. **c** Protein levels of p53, caspase 3(a), PARP and GAPDH in LNCaP cells, which were transiently transfected with pIRES2–EGFP (EGFP) or pCMV–p53 (p53) treated with 0, 0.5, and 1 μM of CPT for 24 h, was analyzed by immunoblotting. **d** Protein levels of p53, p21, PARP, and GAPDH in PC3 cells, which were transiently transfected with pIRES2–EGFP (EGFP) or pCMV–p53 (p53) treated with 0, 0.5, and 1 μM of CPT for 24 h, was analyzed by immunoblotting. Quantitative results of immunoblot image are shown on the right side. Caspase 3(a): the activation form of caspase 3. PARP(f): the full length form of PARP. PARP(c): the cleaved form of PARP. **p < 0.01 vs control

The two most effective chemotherapy agents to generate genotoxic stress are DOX and CPT. Our previous study indicated that p53-mediated apoptosis occurs in response to DOX-induced genotoxic stress in prostate cancer^16^. Here, we asked if p53-mediated apoptosis also occurs with genotoxic stress induced by CPT. CPT efficiently induced apoptosis in a dose-dependent manner in LNCaP cells, which have WT *p53* (Fig. 1a). Interestingly, p21 expression went through the opposite route of caspase 3 activation (Fig. 1a). Furthermore, we scrutinized CPT effect on *p53*-null PC3 cells. We could not detect the significant activation of caspase 3 and only showed PARP full length (f) and its cleaved (c) form in response to various concentrations of CPT (Fig. 1b). By using the ratio of PARP(c) to PARP(f) to define the effect of apoptosis, we showed that apoptosis initiates at 0.5 μM and saturates at 1 μM (Fig. 1b). Increasing CPT concentration could not affect the extent of apoptosis, suggested that CPT has no capacity to maximize apoptosis in this *p53*-null PC3 cell.

Then we assessed if CPT-induced apoptosis is regulated by p53 with overexpression of p53 in LNCaP and PC3 cells. The result clearly indicated that overexpression of p53 increases CPT-induced apoptosis in LNCaP, not in PC3 (Fig. 1c, d). The over-expressed p53 activated p21 expression without CPT, while the p21 expression induced by CPT was independent of p53 in PC3 (Fig. 1d). We speculated that severe DNA damage induced by either DOX or CPT might produce similar genotoxic stress to activate p53 function and enhance apoptosis, and the other factor, which is unavailable in PC3, might involve in this action. However, p21 expression induced by DOX or CPT was p53-dependent or -independent, respectively.

### BTZ-induced non-genotoxic stress activated p53-mediated apoptosis

We further explored whether stress other than genotoxicity can also activate p53-mediated apoptosis.The proteasome belongs to the ubiquitin-proteasome family, responsible for degrading about 90% of intracellular proteins^19^. BTZ is a 26 S proteasome inhibitor approved by the Food and Drug Administration and European Medicine Agency for the treatment of multiple myeloma and mantle cell lymphoma^20^. Unlike DOX and CPT, which generate genotoxic stress, BTZ triggers mainly endoplasmic reticulum stress and unfolded protein response, consequently causing apoptosis^21^. We therefore asked if p53 can facilitate BTZ-induced apoptosis in prostate cancer.

**Fig. 2:**
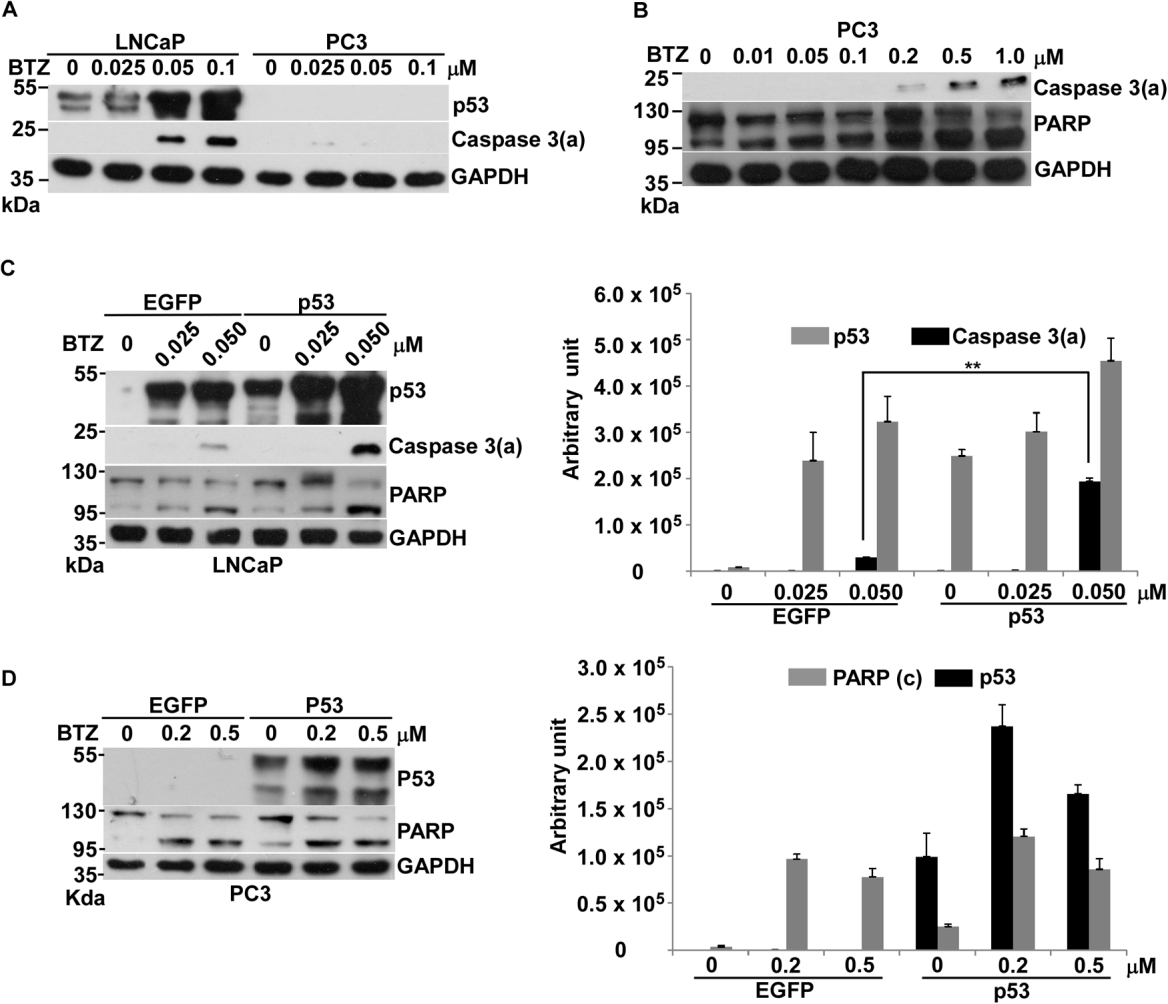
Effects of BTZ on apoptosis in LNCaP or PC3 cells. **a** Comparison of protein levels of p53, and caspase 3(a) and GAPDH, between LNCaP and PC3 cells treated with 0, 0.025, 0.05, and 0.1 μM of BTZ for 36 h, analyzed by immunoblotting. **b** Protein levels of caspase 3(a), PARP and GAPDH in PC3 cells treated with 0, 0.01, 0.05, 0.1, 0.2, 0.5, and 1 μM of BTZ for 36 h analyzed by immunoblotting. **c** Protein levels of p53, caspase 3(a), PARP and GAPDH in LNCaP cells, which were transiently transfected with pIRES2–EGFP (EGFP) or pCMV–p53 (p53) treated with 0, 0.025, and 0.05 μM of BTZ for 36 h, analyzed by immunoblotting. **d** Protein levels of p53, PARP and GAPDH in PC3 cells, which were transiently transfected with pIRES2–EGFP (EGFP) or pCMV–p53 (p53) treated with 0, 0.025, and 0.05 μM of BTZ for 36 h, analyzed by immunoblotting. Quantitative results of immunoblot image are shown on the right side. **p < 0.01 vs control

Our results first revealed that the concentration of BTZ to drive apoptosis for *p53* WT LNCaP cells is 10-folder lower than that for *p53*-null PC3 cells, suggesting that p53 might play a role in BTZ-induced apoptosis (Fig. 2a, b). After overexpression of p53, BTZ-induced apoptosis significantly increased in LNCaP, not in PC3 cells (Fig. 2c, d). This result indicated that p53 has an essential role in BTZ-induced apoptosis in prostate cancer. Since the cellular stress caused by BTZ does not affect the integrity of the genome, we considered this to be non-genotoxic stress. Thus, we thought that p53 can also sense non-genotoxic stress to drive apoptosis in LNCaP cells. However, the other factor, which involve in this p53-mediated apoptosis, maybe absent in PC3 cell.

### p53-mediated apoptosis in response to DOX- or CPT-induced genotoxic stress was transcription-independent, while p53-mediated apoptosis for BTZ-induced non-genotoxic stress was transcription-dependent in prostate cancer

By using a p53DN mutation, p53mt135, which can repress the transcription activity of wild-type p53^22^, we establish a stable cell line, LNCaP–p53DN^23^. Our previous work indicates that DOX-induced p21 expression is significantly reduced in LNCaP–p53DN compared to parental LNCaP cells^23^. Here we used LNCaP–p53DN in comparison with LNCaP–EGFP to ask if p53-mediated apoptosis occurs through its transcription action in response to genotoxic stress and non-genotoxic stress.

**Fig. 3:**
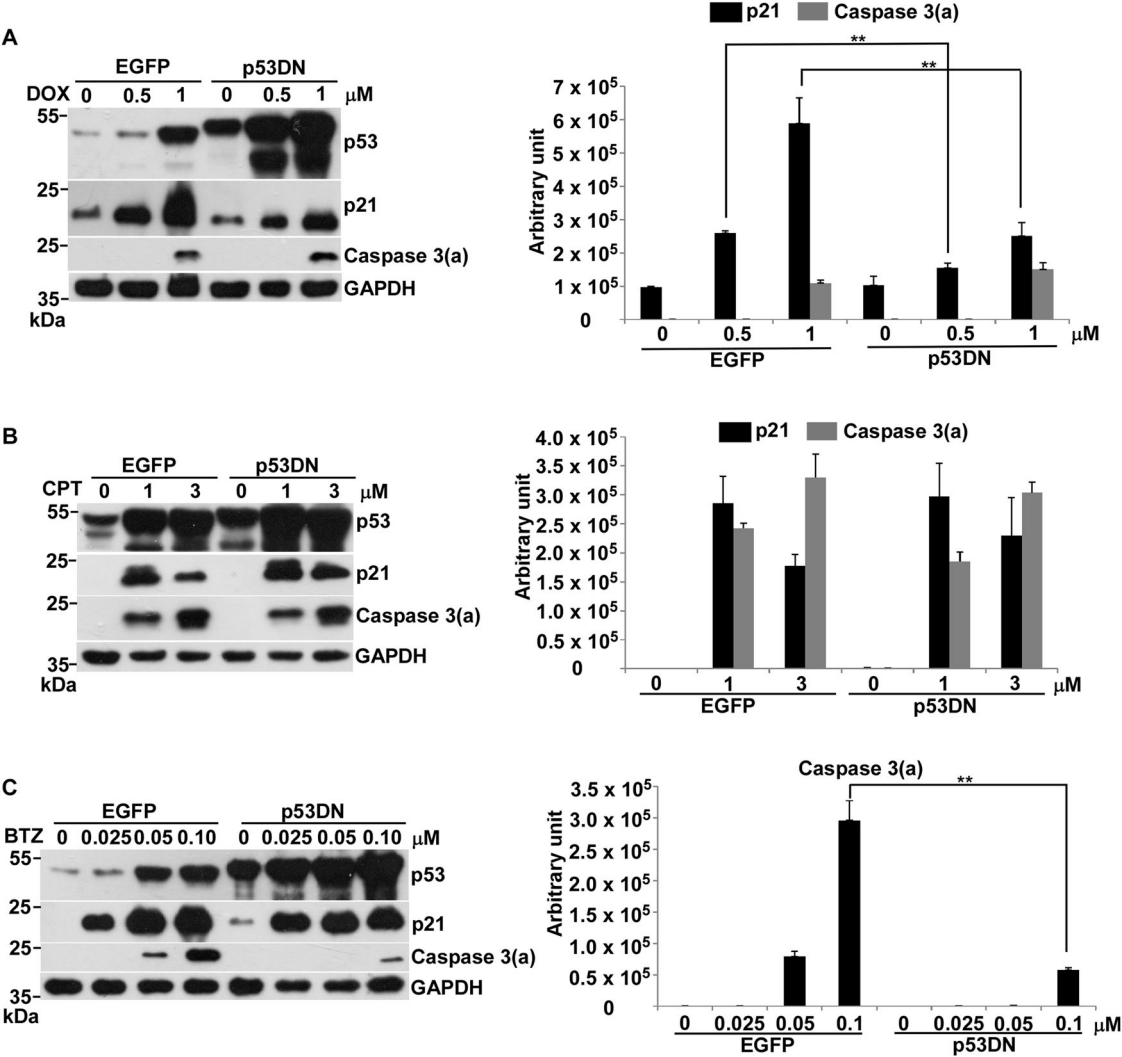
Effects of p53DN on transcription activity and apoptosis in LNCaP cells in response to DOX, CPT and BTZ. **a** Comparison of protein levels of p53, p21 and caspase 3(a) and GAPDH between LNCaP–EGFP (EGFP) and LNCaP–p53DN (p53DN) cells treated with 0, 0.5, and 1 μM of DOX for 24 h, analyzed by immunoblotting. **b** Comparison of protein levels of p53, p21 and caspase 3(a) and GAPDH between LNCaP–EGFP and LNCaP–p53DN cells treated by 0, 1, and 3 μM of CPT for 24 h, analyzed by immunoblotting. **c** Comparison of protein levels of p53, p21 and caspase 3(a) and GAPDH between LNCaP–EGFP and LNCaP–p53DN cells treated with 0, 0.025, 0.05, and 0.1 μM of BTZ for 36 h, analyzed by immunoblotting. Quantitative results of immunoblot image are shown on the right side. **p < 0.01 vs control

Our results first demonstrated that DOX-induced p21 expression was repressed by p53DN by more than 50% (Fig. 3a), suggested that it is transcriptionally activated by p53 for responding DOX and is consistent with our previous publication^23^. Different from DOX, CPT-induced p21 expresssion was not affected by p53DN (Fig. 3b), advocated that it might be p53-independent and is persistent with the above result (Fig. 1d). Significantly, DOX- or CPT-induced apoptosis was not affected by p53DN (Fig. 3a, b), suggested that p53-mediated apoptosis in response to genotoxic stress is transcription-independent. In contrast, the apoptosis induced by BTZ was significantly inhibited by p53DN, indicated that this p53-mediated apoptosis might be transcription-dependent (Fig. 3c).

### The translocation of p53 from cytoplasm to nucleus still actively proceeds during apoptosis in response to genotoxic stress

Our results showed that p53-mediated apoptosis in response to genotoxic stress induced by DOX or CPT is mainly transcription-independent. Unlike genotoxic stress, the non-genotoxic stress induced by BTZ was transcription-dependent. Previous studies have claimed that the interaction of p53 with members of the Bcl2 family in cytoplasm represents an alternative apoptotic pathway^24^. Thus, we asked if the localization of p53 corresponds to its function either in the cytoplasm or the nucleus to activate apoptosis in a transcription-independent or transcription-dependent manner.

**Fig. 4:**
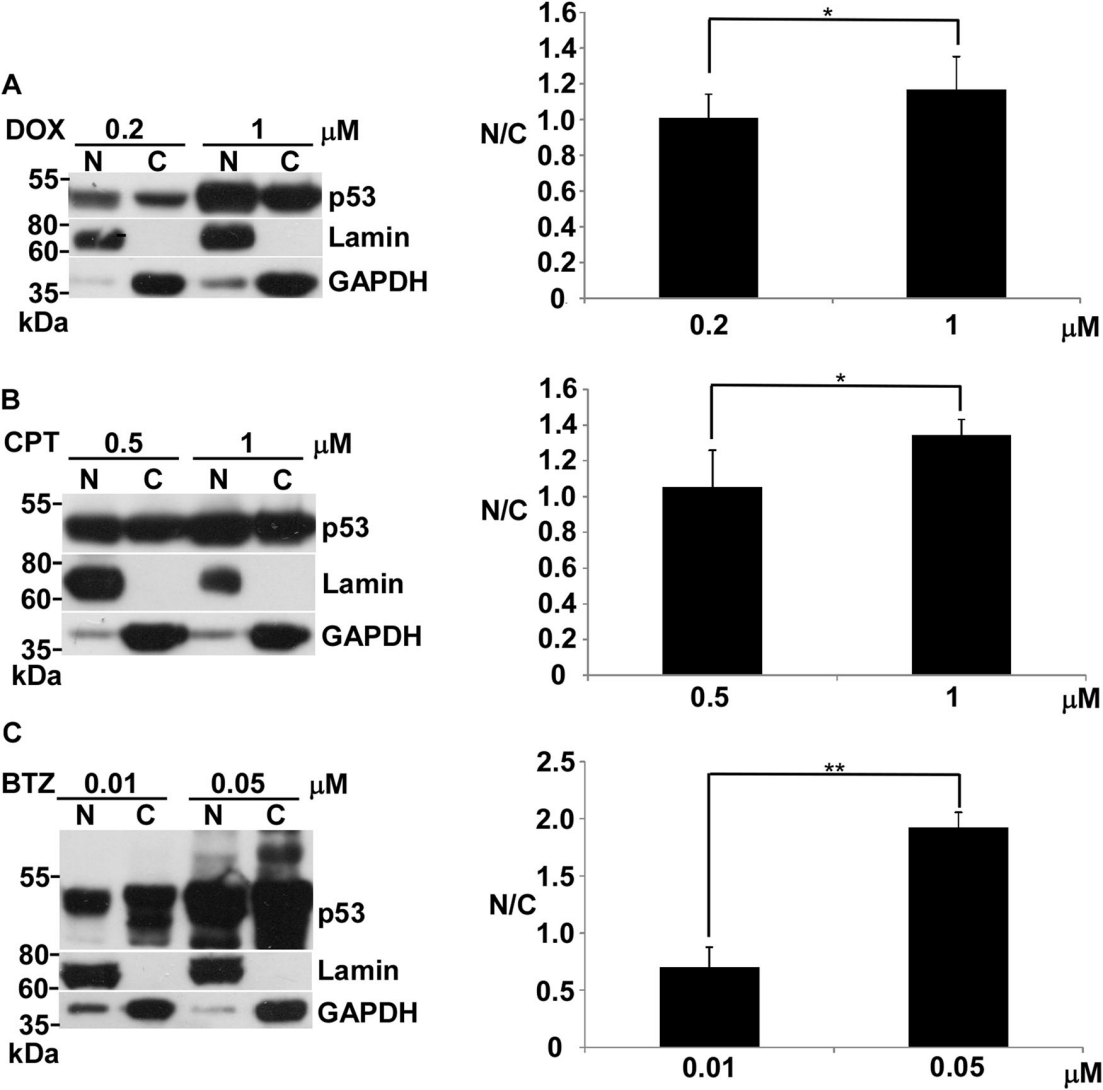
Effects of DOX, CPT or BTZ on nuclear localization of p53 in LNCaP cells. **a** Protein levels of p53, lamin, and GAPDH in nucleus and cytoplasm in LNCaP cells treated with 0.2 and 1 μM of DOX for 24 h were analyzed by immunoblotting. **b** Protein levels of p53, lamin and GAPDH in nucleus and cytoplasm in LNCaP cells treated with 0.5 and 1 μM of CPT for 24 h was analyzed by immunoblotting. **c** Protein levels of p53, lamin, and GAPDH in nucleus and cytoplasm in LNCaP cells treated with 0.01 and 0.05 μM of BTZ for 36 h was analyzed by immunoblotting. The quantitative ratio of nucleus (N) to cytosol (**c**) is shown on the right side. *p < 0.05 vs control, **p < 0.01 vs control

We used the nucleus/cytosol fraction to examine the distribution of p53 between the nucleus and cytosol in apoptosis or non-apoptosis conditions. A slight increase in the p53 ratio of nucleus to cytosol along with increasing apoptosis was seen in DOX- and CPT-treated cells, in comparison with non-apoptosis conditions (Fig. 4a, b). For the BTZ-treated cells, the p53 ratio of nucleus to cytosol increased significantly under apoptosis vs non-apoptosis (Fig. 4c).

## Discussion

Our previous study showed that the BH3-ony protein Bim counteracts Bcl-xl directly to initiate apoptosis in prostate cancer cells^23^. Over-expression of p53DN represses the transcription activity of p53 for p21 expression, but it has no effect on apoptosis in prostate cancer cells in response to DOX^23^. This study indicates that Bim’s initiation of apoptosis caused by DOX is p53-independent in prostate cancer. The role of p53 can be distinguished into two events by the extent of genotoxic stress induced by DOX^16^. Accordingly, p53 activates cell cycle arrest or apoptosis in response to low or high genotoxic stress, respectively. Here, we further found that p53 functions as a transcription factor or as a regulator to activate p21 expression in low genotoxic stress or to escalate to apoptosis in a transcription-independent manner in high genotoxic stress, respectively, in response to DOX. Another DNA damage agent, CPT, could drive p21 expression in p53-independent manner, whereas it induced apoptosis through p53 in transcription-independent manner in high genotoxic stress. A recent study addresses that CPT-induced p21 expression is independent of p53 in human myeloid leukemia cells^25^, consistent with our result. NFκB plays an essential role of CPT-induced p21 expression in human myeloid leukemia cells. Whether CPT-induced p21 expression is regulated by NFκB is another interesting issue to be worthily pursued.

p53 can promote an alternative apoptotic pathway, which contributes a small part of total apoptotic outcome, by interacting with several members of the Bcl2 family in cytoplasm in a transcription-independent manner^24^. Our results showed that the p53 transcription-independent pathway represents the major part of the apoptotic response to high genotoxic stress. Thus, the transcription-independent action of p53 in genotoxic stress-induced apoptosis appears not to go through the same apoptosis mechanism in cytoplasm. Our results showed that the translocation of p53 into the nucleus still actively proceeds even in heavy apoptosis conditions (Fig. 4a, b). This implies that p53-regulated molecular events other than transcription happen in the nucleus to cause apoptosis. Possibly severe genotoxic stress occurring in the nucleus might generate a different signal from the signal driven by low genotoxic stress, resulting in transcription-independent p53-driven apoptosis. How p53 triggers this transcription-independent programmed cell death is an important issue that needs to be pursued to really resolve the mechanisms p53-mediated apoptosis under high genotoxic stress.

In addition, we showed that BTZ, a 26 S proteasome inhibitor, not only stabilizes p53 but also activates p53 transcriptional capacity to enhance apoptosis in response to BTZ’s non-genotoxic stress. By stabilization of BH3-only proteins p53, p21, and p27 and the upregulation of c-Jun-NH_2_ terminal kinase and the downregulation of inhibitor of κBα, BTZ represses cell proliferation and induces apoptosis in several types of cancer^21^. In terms of how p53 function is affected by BTZ, two respective studies found that BTZ acts independently of p53 and induces cell death via apoptosis in B-cell lymphoma and several human cancer cell lines^26,27^, inconsistent with our results. Indeed, BTZ could drive PC3 cells, which contain a *p53*-null gene, toward apoptosis in high concentration of about 0.5 μM, about 10-fold higher than that for LNCaP (Fig. 2b). This suggested that p53 might not function to initiate apoptosis but still enhances apoptosis with severe cellular stress. This may be a reason why the apoptosis caused by BTZ appears to be p53-independent proposed by other studies^26,27^. Unlike p53 effects in genotoxic stress, p53 stabilized by BTZ might transcriptionally activate target genes that are specific to non-genotoxic stress to enhance apoptosis. Disclosure of these genes will provide new insights into the complex apoptotic mechanisms regulated by p53.

In conclusion, our study disclosed two distinct apoptotic pathways regulated by p53 in response to genotoxic and non-genotoxic stress, in a transcription-dependent and -independent manner, respectively. These findings open a gate to a more refined understanding of the mechanisms of p53-mediated apoptosis in prostate cancer. Whether the pathways discovered here are also found in other types of cancers remains to be explored.

## Materials and methods

### Compounds and plasmids

DOX (Merck Millipore), CPT (Merck Millipore), BTZ (Selleck), and G-418 sulfate (Merck Millipore) were purchased as indicated. The two plasmids, pCMV-p53 (Clontech) and pIRES2–EGFP (Clontech), were purchased as indicated.

### Cell culture and compound treatment

LNCaP and PC3 prostate carcinoma cell lines were obtained from the Bioresource Collection and Research Center (BCRC) in Taiwan. Culture conditions for both cells were 37 °C under 5% CO_2_ in RPMI 1640 medium with 10% fetal bovine serum. About 5 × 10^5^ cells of LNCaP or PC3 were plated on petri dishes (10 cm). When cell growth reached 70–80% confluence, fresh medium was substituted and the cells were incubated with various concentrations of DOX, CPT, or BTZ for 24 h or 36 h. After treatment the cells were harvested, washed with PBS, and spun down.

### Cell transfection and generation of stable lines

For transfection of LNCaP cells, five plates (10 cm) of LNCaP cells at 70% confluence were collected and resuspended in 0.8 ml of serum-free RPMI 1640 medium. Then 0.8 ml of cells were aliquoted into two Gene Pulser Cuvetts (Bio-Rad) each containing 0.4 ml of cells. Then 5 μg of pCMV–p53 or pIRES2–EGFP were added to each cuvette. The cells in the cuvette were electroporated by Bio-Rad Gene Pulser at 230 volts and 960 μFaraday. Following transfection, the cells were collected, washed, and plated onto three plates (10 cm) and incubated with RPMI 1640 media containing 10% FBS for 2 days. Then the cultured medium was replaced by fresh medium with various concentrations of CPT for 24 h or BTZ for 36 h and the cells were harvested for immunoblotting. For transfection of PC3 cells, PC3 cells were seeded at 5 × 10^5^ cells per petri dish (10 cm) in 10 ml RPMI 1640 medium with 10% fetal bovine serum and were grown at 37 °C under 5% CO_2_. When cell growth was up to 50% confluence, the old medium was replaced with the fresh medium to incubate for 12 h. Mix 26 μl of FuGENE 6 Transfection Reagent (Permega) with 340 μl of serum-free medium, and incubate for 5 min. Add 5 μg of pCMV–p53 or pIRES2–EGFP into FuGENE 6/medium mixture, and incubate for 15 min. Add the above solution into the cells (10 cm dish, about 70% confluence) dropwise. Culture the transfection cells for 48 h. Then the cultured medium was replaced by fresh medium with various concentrations of CPT for 24 h or BTZ for 36 h and the cells were harvested for immunoblotting. To generate the LNCaP–EGFP stable line, pIRES2–EGFP plasmid DNAs were transfected into LNCaP cells by electroporation as described above. The transfected cells were grown in medium containing 500 μg/ml G-418 sulfate for 3 weeks, and then the resistant colonies, regarded as the stable clones, were picked for immunofluorescence validation by microscope. LNCaP–EGFP and LNCaP–p53DN were cultured in medium with 500 μg/ml G-418 sulfate. When cell growth reached 70–80% confluence, fresh medium without G-418 sulfate was substituted and the cells were incubated with various concentrations of DOX, CPT, or BTZ for 24 h or 36 h. After treatment the cells were harvested, washed with PBS, and spun down.

### Cytosol and nuclear fractionation

Three plates of LNCaP cells treated by BTZ, CPT, or DOX were harvested and then washed in hypotonic buffer (10 mM Hepes pH 7.9, 1.5 mM MgCl_2_, 10 mM KCl, 0.5 mM DTT) with protease and phosphatase inhibitors. The washed cell pellets were resuspended in hypotonic buffer for 10 min to swell cells. The swollen cells were homogenized by 5 up-and-down pushes through the syringe with a 26 1/2 needle. The nuclei were spun down by centrifuging for 15 min at 4000 rpm. After spinning down, the cytosol supernatant and the collected nuclei were lysed in RIPA buffer (25 mM Tris-HCl pH 7.6, 150 mM NaCl, 1% NP40 1 mM DTT, 0.1% NP-40, 1% sodium deoxycholate, 0.1% SDS) containing protease and phosphatase inhibitors. Both cytosol and nuclear lysates were analyzed by immunoblotting.

### Immunoblotting

The harvested cells were lysed in RIPA buffer containing protease and phosphatase inhibitors. The protein concentrations from the cell lysate, separated cytosol and nuclear lysate were determined by BCA Protein Assay Kit (Pierce). About 60 μg of protein per well was subjected to SDS-PAGE. After electrophoresis, the proteins were transferred to a nitrocellulose membrane. The transferred membranes were blocked in 5% (w/v) nonfat dry milk or 5% (w/v) BSA in TBS (0.5 M NaCl, 20 mM Tris-HCl, pH 7.4) with 0.1% (v/v) Tween 20 and probed for the first antibody, followed by incubation with a secondary antibody conjugated with horseradish peroxidase (Cell Signaling) with visualization by ECL (Merck Millipore) with photographic film development. The first antibodies used in this study were anti-GAPDH (Cell Signaling, #5174), anti-caspase 3(a) (Cell Signaling, #9661), anti-PARP (Cell Signaling, #9542), anti-p21 (Cell Signaling, #2947), anti-lamin A/C (Cell Signaling, #4777), and anti-p53 (Santa Cruz Biotechnology, Sc-126). Immunoblot images were quantitated by Image Studio Lite (LI-COR Biosciences).

### Statistical analysis

A paired *t*-test was used to show the statistical significance of the results using JMP13. *p < 0.05 or **p < 0.01 was considered significant.
